# Passive smoking, invasive meningococcal disease and preventive measures: a commentary

**DOI:** 10.1186/1741-7015-10-160

**Published:** 2012-12-10

**Authors:** Harunor Rashid, Robert Booy

**Affiliations:** 1 National Centre for Immunisation Research and Surveillance of Vaccine Preventable Diseases, The Children's Hospital at Westmead and The University of Sydney, New South Wales, Australia; 2 Sydney Emerging Infections and Biosecurity Institute, The University of Sydney, New South Wales, Australia

**Keywords:** Conjugate meningococcal vaccine, invasive meningococcal disease, meningococcal carriage, passive smoking, quitting ratio

## Abstract

Active smoking is a recognized risk factor of various infectious diseases. In a systematic review published in BMC Public Health, Murray *et al. *demonstrated that exposure to passive smoking significantly increased the risk of meningococcal disease among children. Their review especially highlights that the risk remains high even if the exposure occurs during pregnancy or after birth, although the authors could not disentangle the independent effects of smoking during pregnancy from those in the postnatal period. How passive smoking increases the risk of childhood meningococcal disease is not precisely known. Both exposure to 'smoke', or 'smokers' (who are highly susceptible to pharyngeal carriage of meningococci) are postulated mechanisms, but unfortunately very few studies have examined the risk of exposure by considering these two variables separately, and this therefore remains a research priority. Quitting may well be the mainstay of preventing tobacco-related hazards but the available global data suggest that most smokers are reluctant to quit. Among other interventions, immunizing children with a meningococcal conjugate vaccine could, theoretically, reduce the risk of meningococcal disease among children and their smoker household contacts through herd immunity.

See related article http://www.biomedcentral.com/1471-2458/12/1062

## Commentary

A World Health Organization estimate suggests that tobacco kills about 6 million people worldwide annually with one tenth of these being non-smokers exposed to second-hand smoke [1]. The effect of cigarette smoke on various organs and systems of the body, ultimately potentiating the risk of infection, is also well-recognized. Exposure to tobacco smoke reduces neutrophil phagocytic ability, depresses cytotoxic effects of natural killer cells, decreases secretory immunoglobulin A, induces oxidative stress and adversely affects immune responses, including host response to vaccination [2].

The passive coating of buccal epithelial cells with components of cigarette smoke can enhance binding of potentially pathogenic bacteria, including meningococci. A systematic review by Murray *et al. *published in BMC Public Health has shown that household passive smoking significantly increased the risk of meningococcal disease in children [3]. This is consistent with at least one other systematic review published recently that demonstrated that not only the risk of meningococcal disease but also the incidence of carriage was increased in children who were exposed to second-hand smoking [4]. However, Murray and colleagues noted that exposure of unborn or newborn children to maternal smoking also increased the risk of meningococcal disease in infants [3].

But how does the risk of meningococcal disease increase in children exposed to passive smoking? One line of reasoning is that the increased risks of meningococcal disease and carriage are due to the effect of exhaled smoke. This view is supported by observations that environmental exposure to other smokes (for example, kitchen smoke) can increase the risk of meningococcal disease. A case-control study to investigate possible risk factors among survivors of a meningitis epidemic in 1997 in northern Ghana demonstrated that cooking in kitchens with firewood stoves increased the risk of meningococcal meningitis nine-fold [5].

There is also evidence that contact with smokers, irrespective of whether they smoke within the household milieu, can increase risk. For instance, in a case-control study examining whether there were medical and/or environmental factors associated with meningococcal carriage, Stuart and colleagues demonstrated that active smoking and the presence of other smokers were independently associated with meningococcal carriage [6]. The authors explained that the higher risk of carriage in those who live with smokers may be due to the greater chance of acquiring meningococci from contact with smokers and 'not to a direct effect of passive smoking.' This notion is also supported in respect of meningococcal disease by prospective population-based case-control studies conducted in Australia and the UK [7-9]. An Australian study showed that having a smoker amongst close contacts increased the odds of having meningococcal disease by 3.7 times [7], while one UK study showed that the odds ratios increased with the number of smokers in the household [8], and another UK study indicated that the principal risk attributable to passive smoking was in fact due to exposure to the smoker rather than the smoke [9].

Additionally, there is evidence that smoking outside the home only, as reported by parents, does not reduce nicotine levels in the hair of children, possibly indicating that smokers continue to exhale nicotine after the actual time of smoking [10]. These studies suggest that the risk of meningococcal disease among contacts of smokers cannot be reduced by simply banning smoking in confined places like houses, work places, hospitals and vehicles, but that a total ban is required.

But, all these studies are liable to bias as the information about smoking status given by parents can at times be misleading or 'deceptive.' More objective studies, for example by the use of cotinine measurements, would help to explore the association of meningococcal disease with passive smoking. Also, whenever possible, future studies should aim to tease out exposure to 'passive smoke' from exposure to 'smoker contact,' so that the role of smokers in increasing the risk of meningococcal disease in household contacts can be understood more clearly.

The study by Murray and colleagues highlights the importance of stopping smoking for parents [3]. But in a recently published large survey representing 3 billion people from 16 countries, quitting ratios have been found to be very low (< 20%) [11]. Other interventions like health education, close monitoring of children for meningococcal disease, tighter legislation (including plain packaging) and targeted interventions for quitting smoking in pregnancy could be employed. Vaccines may play an important role by safeguarding household contacts of smokers from contracting serious vaccine-preventable diseases. But, ironically, despite a recognized association between smoking and infection [2], smokers are not generally considered as a risk group requiring vaccination. One exception is the recent recommendation of the US Advisory Committee on Immunization Practices for 23-valent pneumococcal polysaccharide vaccine in smokers aged 19 years or older [12]. But that recommendation is primarily focused on prevention of invasive pneumococcal disease in smokers, not their household contacts.

Immunizing children with a pneumococcal conjugate vaccine has had enormous herd benefits for their parents and grandparents [13]. Similarly for meningococcal disease, vaccinating children against serogroup C and, more recently, against serogroup A diseases has produced significant herd benefits to adults [14,15]. The study by Murray and colleagues adds to a large body of evidence suggesting that the risk of meningococcal disease in children increases on exposure to second-hand smoking and/or smoker contacts. Therefore, vaccinating children with conjugate meningococcal vaccines (as well as the newly licensed meningococcal B vaccine) could be an appropriate strategy to protect the children, and hopefully also their adult smoker contacts (through herd immunity). Nevertheless, further studies are needed to evaluate the cost-effectiveness of such a strategy.

## Competing interests

RB has received financial support from pharmaceutical companies CSL, Sanofi, GSK, Novartis, Roche and Pfizer to conduct influenza control research and attend and present at scientific meetings. Any funding received is directed to a National Centre for Immunisation Research and Surveillance of Vaccine Preventable Diseases (NCIRS) research account at The Children's Hospital at Westmead and is not personally accepted by Professor Booy. The other authors have no conflict of interest to declare.

## Authors' contributions

RB conceived the idea, HR wrote the first draft, RB modified and contributed to the subsequent versions. Both authors approved the final version of the manuscript.

## Authors' information

HR is a clinical research epidemiologist at the NCIRS, Australia. He is particularly interested in the epidemiology of vaccine-preventable infections among travelers, including meningococcal disease and respiratory infections.

RB is currently the Head of Clinical Research at NCIRS, Australia. He has particular interests in meningococcal and pneumococcal disease, Hib, influenza, varicella and HPV. His research interests extend from understanding the genetic basis of susceptibility to, and severity of, infectious diseases, especially influenza and invasive disease caused by encapsulated organisms; the clinical, public health, social and economic burden of these diseases; and means by which to prevent or control serious infections through vaccines, drugs and non-pharmaceutical measures.

## Pre-publication history

The pre-publication history for this paper can be accessed here:

http://www.biomedcentral.com/1741-7015/10/160/prepub
